# Cerebrocerebellar system and Arnold’s bundle - A tractographic study: preliminary results

**DOI:** 10.1590/S1980-57642010DN40400007

**Published:** 2010

**Authors:** Eliasz Engelhardt, Denise Madeira Moreira, Jerson Laks

**Affiliations:** 1 Cognitive and Behavioral Neurology Unit – INDC/CDA/IPUB-UFRJ.; 2 Neuroradiology Unit – INDC – UFRJ.; 3 Center for Alzheimer Disease/CDA/IPUB – UFRJ.; 4 PróCardíaco Hospital Radiology Service – RJ.

**Keywords:** tractography, frontopontine bundle, Arnold’s bundle, cerebrocerebellar system

## Abstract

The cerebellum, traditionally considered a structure involved in balance and movement control, was more recently recognized as important in cognitive, emotional and behavioral functions. These functions appear to be related to the more recent parts of the cerebellum that belong to the cerebrocerebellar system. One of the key segments of this system is the (pre)fronto-[penduncule]-pontine projection that represents the Arnold’s bundle. Diffusion tensor imaging and tractography (DTI-TR) has permitted in vivo virtual dissection of white matter tracts, including those of the cerebellar.

**Objective:**

To study the fronto-[peduncule]-pontine projection (Arnold’s bundle), with DTI-TR.

**Methods:**

Ten normal subjects were included (mean age 30 years). Standard acquisitions in three planes were obtained with a 1.5T GE Signa Horizon scanner, complemented with DTI acquisitions. Post-processing and analysis was performed using an ADW 4.3 workstation running Functool 4.5.3(GE Medical Systems). A single ROI was placed on the medial third of the cerebral peduncle base, considered the site of convergence of the fibers of Arnold’s bundle, bilaterally.

**Results:**

Twenty tractograms were obtained. All were constituted by a significant number of fibers in correspondence to the frontal lobe, and part of them anterior to the coronal plane at the anterior commissure, which characterizes them as associated to the prefrontal region.

**Conclusions:**

For the first time, frontal lobe related projections were systematically revealed with DTI-TR seeded from cerebral peduncle base ROIs. They showed anatomic coherence with Arnold’s bundle, which includes the prefrontopontine segment of the cortico-ponto-cerebellar path, one of the components of the cerebrocerebellar system, acknowledged as fundamental for non-motor functions such as cognition, emotion and behavior.

## Abstract (pt)

O cerebelo, tradicionalmente considerado como estrutura relacionada ao equilíbrio e controle do movimento, foi mais recentemente reconhecido ser importante em funções cognitivas, emocionais e comportamentais. Tais funções encontram-se relacionadas às partes mais novas do cerebelo que pertencem ao sistema cerebrocerebelar. Um dos segmentos de relevo desse sistema é a projeção (pré)fronto-[pedúnculo]-pontina que representa o feixe de Arnold. A imagem com tensor de difusão e a tratografia (DTI-TR) vem possibilitando a dissecção virtual de feixes da substância branca, inclusive os cerebelares.

**Objetivo:**

Estudar a projeção fronto-[pedúnculo]-pontina (feixe de Arnold), com DTI-TR.

**Métodos:**

Foram incluídos dez sujeitos normais (média de idade 30 anos). Sequências padrão em tres planos foram obtidas com equipamento Signa Horizon GE de 1,5T e complementadas com aquisições de DTI. Pós-processamento e análise foram realizados em uma estação de trabalho ADW 4.3 com Functool 4.5.3(GE Medical Systems). Foi utilizado ROI único colocado no terço medial da base do pedúnculo cerebral, considerado o local de convergência das fibras do feixe de Arnold, bilateralmente.

**Resultados:**

Foram obtidos vinte tratogramas. Todos eram constituídos por um número significativo de fibras em correspondência ao lobo frontal e parte dessas em posição anterior ao plano coronal à comissura anterior, que as caracteriza como relacionadas à região pré-frontal.

**Conclusões:**

Foram mostradas, pela primeira vez de modo sistemático, projeções relacionadas ao lobo frontal com DTI-TR através de ROIs colocados na base do pedúnculo cerebral. Apresentavam coerência anatômica com o feixe de Arnold, que compreende o segmento pré-frontopontino da via córtico-ponto-cerebelar, um dos componentes do sistema cerebrocerebelar, considerado subjacente a funções não motoras, como cognição, emoção e comportamento.

The cerebellum, traditionally considered a structure involved in balance and movement control,^1-3^ is now also recognized as playing an important role in cognitive, emotional and behavioral functions,^3-7^ and is also implicated in neuropsychiatric disorders.^8^ These more recently studied functions (and dysfunctions) are ascribed to the integrative activity of the cerebrocerebellar system constituted by distinctive brain regions and related fiber pathways. Association areas of the cerebral cortex and neocerebellar structures (cortical and subcortical) are interconnected. The cortical efferents constitute the corticopontine and mossy fiber projections to the cerebellum. Reciprocally, the cerebellum (dentate nucleus) projects to cortical association areas via cerebellothalamic and thalamocortical pathways.^9,10^

Classically, neuroanatomical studies have been performed on post-mortem human specimens, with dissection of normal brains and gross and microscopic tracking of degenerated fiber bundles of brains that had suffered lesions (vascular, traumatic, surgical). Despite the limitations of such studies the main tracts of the central nervous system were fairly accurately demonstrated and described, including the corticopontine group of fibers.^11-19^

The knowledge that the corticopontine-[cerebellar] projections originate from several areas of the cerebral cortex (frontal, parietal, occipital, and temporal) and converge to the homolateral cerebral peduncle base(s) (basis pedunculus[i], pes pedunculus[i]) is not new,^20,13^ although has achieved a higher level of detail only more recently.

The description of these projections into the brain stem (pedunculus, pons, medulla) preceded the elucidation of their cortical origin.^21^ It is presently acknowledged, albeit not fully settled, that the frontopontine tract stems from the prefrontal cortex (Brodmann areas [BA] 10, 9, 8, 45, and 46), containing also fibers from the precentral region (BA 4 and 6), while the cortico-(bulbo)-spinal tract originates from the central region (mainly from BA 4, somesthetic [BA 3, 2, 1], and less from SMA, and BA 6, 8, 5 and 7),^22-28^ and the posterior projections come from the extensive retrorolandic region, including parietal, occipital and temporal areas.^15^

In spite of a long history of investigations, including numerous anatomo-pathological studies in humans and experimental studies in non-human primates,^29-30^ the topographical organization of the fibers at the base of the cerebral peduncle still has some unresolved details concerning its constituent fibers. It is recognized that the frontopontine projection first depicted and described by Arnold (1838)^31^ extends from the frontal cortex through the anterior limb of the internal capsule to the pons, passing through the medial part of the cerebral peduncle base.^12-13^ The lateral peduncle base is occupied by the tract described by Türck (1851), formed by retrorolandic (parieto-occipito-temporo-pontine) projections.^13,15^ The intermediate part is the route of the cortico-(bulbo)-spinal or cortico-(nucleo)-spinal tract, that extends through the internal capsule to caudad levels, and comprises the corticospinal part (pyramidal tract as named by Türck, 1851),^21^ and the corticobulbar tract. Most fibers of the corticobulbar tract accompany the corticospinal fibers through the internal capsule (genual fibers) and separate as they traverse the brainstem. Part of these fibers terminate in the pontine nuclei which originate projections to the cerebellum.^32-33^ The proportion corresponding to each segment varies according to several authors, with the classical trend considering the middle segment to be larger than the medial and lateral ones.^13,34-36^ However, there are few such studies in the human brain, and it should be noted that these have been carried out in brains with natural^13,15^ or surgical^12^ lesions which render them with imprecision, or resulting from post-mortem dissections.^37^ On the other hand, studies with magnetic resonance imaging (MRI) and diffusion tensor acquisition have shown that the distribution of the peduncular fibers is somewhat different in comparison to the classical model, with roughly equal segmentation.^38-43^

The frontopontine bundle (Arnold’s bundle) together with that originating from the posterior cortex (Türck’s bundle) includes numerous fibers of the cortico-ponto-[cerebellar] limb of the cerebrocerebellar system that underlies non-motor functions.^30^ Additionally, clinical and neurophysiologic research in patients with lesions of the cerebellum and/or its pathways have allowed clinicians to identify the presence of significant cognitive and affective disorders,^6^ and recent functional neuroimaging studies substantiate such findings.^44^

Studies with MRI and DTI followed by tractography (DTI-TR) have permitted virtual dissection of white matter tracts, including cerebellar ones, in vivo, in the human brain,^38,41,45-48^ but systematic studies of the cerebrocerebellar system in the human brain are limited, while studies on corticopontine projections are lacking. A preliminary report on this issue was recently presented.^49^

Given the scarcity of tractographic studies on the human cerebrocerebellar system, the aim of this study was to visualize cortico-[peduncle]-pontine projections from (pre)frontal cortex (Arnold’s bundle) with DTI-TR, with the seeding ROIs placed at the level of the cerebral peduncle base.

## Methods

**Figure 1:**
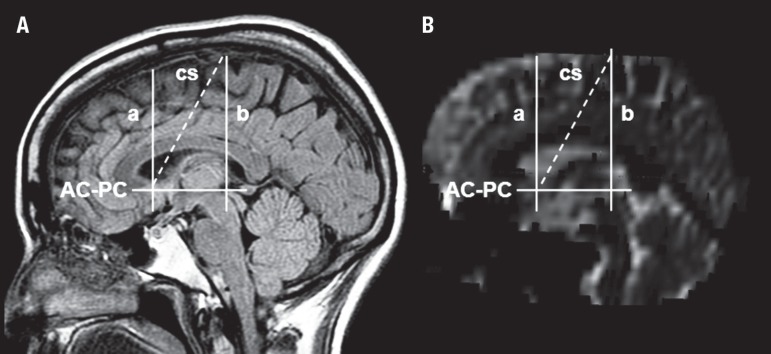
Main markers used to limit prefrontal region. [A] MR sagittal plane and T1 acquisition. [B] Reconstructed sagittal plane from EPI (echo-planar imaging). The prefrontal region is anterior to the anterior commissure plane, and the premotor-motor region is anterior to the central sulcus line (broken line)^52-54^. AC-PC: anterior commissure-posterior commissure line; a: anterior commissure plane; b: posterior commissure plane; cs: central sulcus line (broken line).

**Figure 2:**
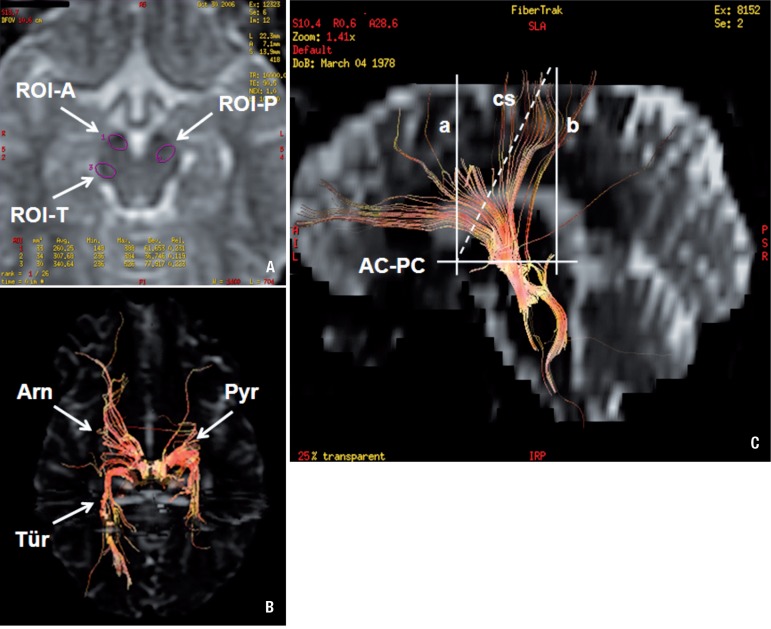
[A] Localization of ROIs at the cerebral pes pedunculi (EPI axial view). [B] Tractograms - axial view. Arrows - right side point to the seeding ROIs and to Arnold’s (Arn) and Türck’s (Tür) bundles, and left side arrows, the seeding ROI and to the cortico-(bulbo)-spinal tract (Pyr). [C] Arnold’s bundle tractogram (sample) - solid line (limit between premotor and prefrontal region) and broken line (projection of the central sulcus - posterior limit of the frontal lobe). AC: anterior commissure; PC: posterior commissure; AC-PC: anterior commissure-posterior commissure line; a: anterior commissure plane; b: posterior commissure plane; cs: central sulcus line.

Ten normal subjects of both genders were included (age 30.67±5.73 years). Standard acquisitions in three planes were obtained with a 1.5T GE Signa Horizon scanner. Diffusion tensor acquisitions, similar to those found in other studies on the issue,^45,50^ were used in the present study with parameters TR/TE=10 000/89.1 msec, matrix=128×128, FOV=30×24 mm, NEX=1, 25 gradient directions, *b*=1000 sec/mm,^2^ slice thickness=5 mm, number of slices=30 without gap, in the axial plane, scan time=4 min, 40 sec. Post-processing and analysis were performed on an ADW 4.3 workstation running Functool 4.5.3(GE Medical Systems). The fiber tracts were reconstructed starting from voxels with an FA>0.18 on the axial plane up to those with an FA<0.18, or up to a maximum step size of 160 µm. A single ROI (of around 40 mm^2^) was placed occupying the medial third of the cerebral peduncle base,^51^ considered the site of convergence of the fibers of the frontopontine tract (Arnold’s bundle), on both sides. Smaller ROIs (of around 5 mm^2^) were placed at more medial and more lateral localizations in the same territory for a better understanding of the topographical distribution of the fibers (Figure 1 for the reference lines, and Figure 2 for the ROIs localization and sample tractograms) The fibers selected with the peduncular ROI originate mainly in the frontal cortex as DTI-TR cannot distinguish between efferent and afferent fibers, and the cerebral peduncles are known to contain fibers that project from the cortex to the pontine nuclei through the pes pedunculus.^41,51^

The present study is part of a larger project on Vascular Cognitive Disorder, approved by the Research Ethics Committee of IPUB-UFRJ. Informed consent was obtained from all participants.

## Results

**Figure 3:**
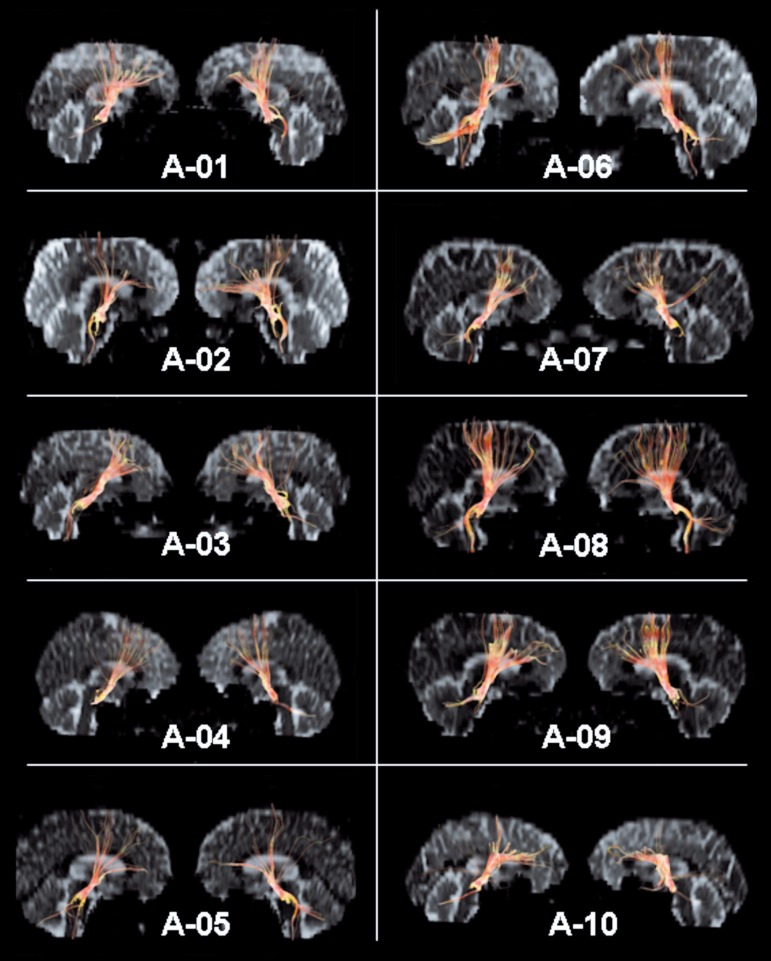
Arnold’s bundle tractograms (right and left) of the 10 subjects (A-01 to A-10).

Twenty tractograms were obtained (Figure 3). The demonstrated fibers tracked from the medial ROI were localized in correspondence to the frontal lobe, anterior to a plane running parallel to the central sulcus.^54^ A significant proportion of these fibers was localized anterior to the coronal plane to the anterior commissure, considered a conservative landmark for the limit between prefrontal and premotor cortex,^53,55^ which characterizes them as associated to the prefrontal region (Arnold’s bundle). The fibers related to more posterior regions of the frontal lobe probably concern premotor and motor areas, which are the main origin of the cortico-(bulbo)-spinal tract, as confirmed by the smaller ROIs. Although the core ROIs were placed practically in the same regions, the tractograms showed intra-subject and inter-subjects variability and asymmetry.

It may be possible that fewer prefrontal fibers than expected were visualized in some subjects. An explanation for this would be a truncation effect as a result of the acute angulation of the frontopontine tract at the level of the cerebral peduncle, in its trajectory from the frontal cortex.^50,56^ On the other hand, the apparent presence of more than the expected number of fibers localized in relation to the premotor-motor region in other subjects may be attributed to a better visualization of less angulated fibers and/or some overlap with perirolandic originating fibers. Individual variations, considering both possibilities, cannot be ruled out.

## Discussion

Projections from the frontal lobe were revealed in the present study with DTI-TR, seeded from cerebral peduncle base medially-placed ROIs. These showed anatomic coherence with the frontopontine bundle (Arnold’s bundle), which includes the prefrontopontine segment of the cortico-ponto-cerebellar path. Several studies containing cerebellar-related tractograms have previously been published,^46,57-59^ but few studies have focused specifically on cortico-peduncular projections as has the present investigation. One of these studies,^57^ for instance, showed the cortical fibers descending to the cerebral peduncle, revealed by placing one ROI around the pes of the cerebral peduncle of one side and the second around the middle cerebellar peduncle on the other side. This approach permitted the whole assembly of fibers that traverse the cerebral pes pedunculus to be visualized, including the cortico-(bulbo)-spinal tract. However, it was unable to show the other two specifically cerebellum-related bundles. Another more detailed study assessed the contributions of cortical areas (prefrontal, motor and posterior parietal) projections to the cerebral peduncle in humans (and macaque monkeys).^41,60^ The cited study showed seven probable distribution maps of the cerebral peduncle, one for each cortical zone, and a clear topographic organization in the cerebral peduncle. Fibers from the prefrontal, primary motor and premotor regions, and from posterior cerebral cortex, occupied almost equal areas, as did those medially placed by the prefrontal fibers. The cited study, according to the authors, adds to the understanding of the corticopontine component of the cortico-ponto-cerebellar system by providing anatomical evidence of a major contribution from the prefrontal cortex to the pontine nuclei.^41^ However, in spite of showing the cortical origin and the peduncular termination of the fibers, the authors did not display the trajectories of the bundles under analysis. Thus, the present study appears to be the first time that the trajectory of the (pre)fronto-[peduncule]-pontine projection is visualized in a systematic study by way of a single ROI placed on its convergence area at the cerebral peduncle base.

The present study showed intra- and inter-subject variability and asymmetry of the obtained tractograms, even though the ROIs were placed in the same location. The variability of the studied tract was previously observed (among subjects, between hemispheres) in a classical study (post-surgical),^12^ and recently by the tractographic technique showing the case-to-case anatomical variability of the peduncular tracts^41^ seen for other tracts.^61-63^ The variability observed in the present study, reflected by different fiber densities comparing the anterior (less than expected?) and more posterior fibers (more than expected?), could also be explained at least in part by a truncation effect due to different degrees of angulation of these fibers.^50,56^

The prefrontal segment of the fronto-[peduncle]-pontine fibers emerges from prefrontal associative cortical areas, and is anatomically related with the evolutionary newer parts of the cerebellum (lateral hemispheric cerebellar cortex and dentate nucleus).^9,60^ Reciprocally, the cerebellum (dentate nucleus) sends fibers out to these cortical areas (dentato-thalamo-frontal projection),^9^ which were also demonstrated tractographically.^38^ These pathways are responsible for the cerebellar’s participation in the neural circuits that underpin cognitive, emotional, and behavioral control.^5,6,10^

Some limitations should be acknowledged. It is possible that fewer prefrontal fibers than expected were visualized because of a truncation effect at the level of the cerebral peduncle due to the acute angulation of the studied tract. On the other hand, the apparent presence of more fibers than expected localized in the posterior frontal region could be attributed to better visualization of less angulated fibers and/or some overlap with perirolandic-derived fibers. Individual variations, in both scenarios cannot be ruled out. Some of these limitations will probably be resolved by future studies aimed at visualizing the projections from (pre)frontal areas to the cerebral peduncle base employing alternative approaches.

## Conclusion

The cerebellum, long considered a motor modulating structure, has more recently been recognized as an important structure underlying cognitive, emotional and behavioral functions. The evolutionary more recent parts of the cerebellum (lateral hemispheric cortex and dentate nucleus), reciprocally connected with associative cortical areas, constitute the cerebrocerebellar system that underpin these functions. One of the key segments of this system is the (pre)fronto-[peduncle]-pontine projection (Arnold’s bundle). The present paper reveals and analyses this bundle with single ROI placement at the medial part of the cerebral peduncle base, and displays it systematically with this approach for the first time.
